# Standby Ties that Mobilize: Social Media Platforms and Civic Engagement

**DOI:** 10.1177/08944393211067687

**Published:** 2022-02-21

**Authors:** Shelley Boulianne

**Affiliations:** Department of Sociology, 3151 MacEwan University, Edmonton, AB, Canada

**Keywords:** Civic groups, civic engagement, donation, volunteer, social media, Instagram, Facebook, Twitter

## Abstract

Nonprofit organizations and groups depend on donations and volunteers for their survival. Digital media can help by offering a platform for making online donations and facilitating online volunteering, but also by identifying and connecting with people who are sympathetic to an organization’s mission. This article employs four-country (USA, UK, France, and Canada) representative survey data (*n* = 6291) to examine the use of social media for establishing connections between citizens and organizations as well as the relationship of these connections to online and offline volunteering and donating. Across all social media platforms considered (Facebook, Instagram, and Twitter), I find significant positive correlations of following nonprofits with online and offline volunteering and donating. However, Facebook has a slightly larger role, which may be attributed to its overall popularity, which can incentivize organizations’ more intense use of this platform.

## Introduction

Citizens can use social media to connect with organizations in their community or across the globe. Once these connections are established, citizens may be mobilized to participate in the organization’s work by donating money or volunteering their time. The Internet facilitates these civic connections, but also provides opportunities to participate in these organizations. Nonprofit organizations and groups depend on donations and volunteers for their survival. The Internet can help mobilize people to participate and offer a platform to donate and volunteer.

Little research has been done on *online* forms of civic engagement, instead focusing on the effects of digital media on *offline* civic engagement (Boulianne, 2020). It is unclear whether the predictors of offline forms are the same as for online forms of civic engagement (Ackermann & Manatschal, 2018). In addition, social media scholarship is increasingly moving toward an analysis of platform-specific effects. Platforms differ in their digital architecture, which affects how citizens and organizations use these tools (Bossetta, 2018). In this paper, I examine civic uses of Facebook, Instagram, and Twitter and their relationship to online and offline volunteering for and donating to nonprofit and charitable organizations. Which social media platforms are being used to cultivate ties to these organizations and to what extent do these connections lead to subsequent donating and volunteering (online, offline)?

This article is unique in three dimensions. First, it examines which social media platforms are used to create these civic connections. I compare Instagram, Facebook, and Twitter, addressing a gap in relation to research on Instagram. I find that Facebook is used more so than other platforms to cultivate ties to civic groups and that Facebook connections have stronger correlations with offline as opposed to online forms of participation. This pattern is explained in terms of Facebook’s popularity with citizens, creating an incentive for civic groups to invest in creating and maintaining their presence on this platform. With so many potential eyes on their page, there is value in offering sophisticated mobilization strategies to increase donating to and volunteering for the organization. In addition, the platform offers more sophisticated targeting and advertising formats compared to Twitter (Bossetta, 2018). This provides an opportunity to target a select group of citizens and advertise on their feeds; targeting subgroups can help tailor mobilization messages to turn sympathizers (followers on social media) into volunteers and donors.

Second, I examine how these connections relate to the likelihood of volunteering and donating as separate and distinct activities, rather than blurring these activities into a single index or scale. Younger people are more likely to volunteer and older people are more likely to donate, compared to other age groups (Turcotte, 2015). I examine whether these differences replicate in the online manifestations of these activities. I offer new insights into age differences in engagement in online and offline forms of donating and volunteering, which illustrate young people are more likely to volunteer online and offline. However, for donating, older people are more likely to engage offline versus online. This is an important contribution as most research on social media and civic engagement focuses exclusively on youth.

Third, the data are from a large sample survey from four countries, providing a robust sample for testing the role of social media use and civic engagement. Most research is based on a single country. This paper focuses on platform-specific outcomes, rather than cross-national differences. My prior research using different data sources suggests no significant differences in the effects of social media on civic participation in different countries (Boulianne, 2019; Boulianne et al., 2019).

## Literature Review

### Platforms

Social networking sites are web-based tools that allow users to create a profile and a network attached to that profile, connect to other profiles, and interact with others (Stoycheff et al., 2017). Social networking sites include Facebook, Twitter, and Instagram. These sites are growing in popularity worldwide; in Western democracies, these sites are used by the majority of citizens (Poushter et al., 2018).

Facebook is the oldest of the platforms, has the largest user base, and has the highest adoption in Western democracies (Newman et al., 2020). Stoycheff et al. (2017) conducted an analysis of Facebook research, concluding little research has been performed on the civic uses and that most research is based on the United States. As such, this article seeks to address this gap, but incorporates a cross-platform perspective. Instagram has more than 1 billion monthly active users (Clement, 2019), yet relatively little research has been conducted on this platform (Blank & Lutz, 2017; Russmann & Svensson, 2016). Instagram is a photo-centric application that was bought by Facebook in 2012. It offers similar engagement tools as Facebook, including the ability to follow civic groups, like their posts, and comment on their posts. Similar to Facebook, groups can pay to promote their message; post lengths are also much longer than allowed on Twitter (Bossetta, 2018). Both Facebook and Instagram offer sophisticated targeting and many advertising formats (Bossetta, 2018). Given the parallels, we might expect similar types of uses and outcomes of use. On the other hand, Instagram is far less popular than Facebook, which might alter the uses and outcomes of this platform (Newman et al., 2020; Perrin & Andersen, 2019). The lower popularity may reduce the motivation for organizations to invest in their presence and activities on Instagram.

Twitter has the smallest user base and lowest penetration compared to the other platforms (Newman et al., 2020; Perrin & Andersen, 2019). Twitter is an elite social media platform, as reflected by the lower adoption levels and smaller user base, but also by who uses it—largely politicians, news media, and celebrities (Newman et al., 2020; Perrin & Andersen, 2019). Compared to Facebook and Instagram, Twitter is distinctive in terms of the short lengths of posts, which may limit its mobilization potential. Similar to Instagram and Facebook, Twitter offers the option to pay to promote content; however, Twitter’s targeting and advertising forms are less sophisticated (Bossetta, 2018).

### Social Media and Civic Ties

Wells & Thorson, 2017 found the strongest relationship between different Facebook uses and civic engagement when they measured “liking” civic and political pages on Facebook. They attribute these strong effects to the role of social media in developing curated flows, which are cultivated by users and their friends as well as computer algorithms and strategic communicators. By liking a civic group’s posts, users are inviting more posts related to that civic group (Wells & Thorson, 2017). The Facebook algorithm responds to this input and adapts the users’ feed accordingly. By following a nonprofit group, citizens are establishing a tie to the organization. This connection suggests the citizen is supportive of the organization and may be willing to engage further. Indeed, they can be viewed as “standby citizens,” a phrase coined to describe how young people are prepared for action by staying alert and keeping informed through news media (Amnå & Ekman, 2014). I adapt the theory as follows: citizens establish these social media linkages to nonprofit organizations so they can stay alert and keep informed; they “are willing and able to participate if needed” (Amnå & Ekman, 2014, p. 262). Citizens are establishing a connection because they support the organization’s goals, but whether they engage further depends on a variety of factors, such as whether they have disposable income, they have free time to volunteer, or the community expresses a need for such engagement.

In terms of research linking citizens’ social media use and engagement in civic and political life, Boulianne (2019) completed a meta-analysis of 133 survey-based research studies. The research clearly establishes a positive relationship between social media use and activities such as protesting, boycotting, and talking politics. Indeed, social media effects may be stronger for civic engagement (e.g., donating, volunteering, and boycotting) than voting and related activities, (Boulianne, 2015). However, compared to other forms of engagement, relatively little research has been done on donating to and volunteering for charitable or community organizations. The meta-analysis affirms a positive relationship, but leaves questions about the magnitude of this positive relationship and differences in civic outcomes for various types of social media platforms. In addition, this research most often employs student or youth samples (Boulianne, 2015, 2019), which raises questions about whether the positive correlations hold for other people.

### Donating versus Volunteering

Wells & Thorson, 2017 sample of Wisconsin students is not helpful for understanding donating and volunteering as distinct activities. Youth and student samples are often used in the study of social media use, donating, and volunteering (Atkinson, 2015; Chan et al., 2016; Kahne et al., 2013; Kim & Khang, 2014; Pasek et al., 2009; Valenzuela et al., 2009; Warren & Wicks, 2011; Wells & Thorson, 2017; Wicks et al., 2013; Xenos et al., 2014; Xie, 2014). This youth-focused research demonstrates positive correlations between social media use and civic engagement. Youth are distinctive in being less likely to donate money but more likely to volunteer. For example, in Canada, less than 30% of youth donate, whereas 66% volunteer (Turcotte, 2015). Furthermore, across the globe, youth and students are more likely to use social media than older age groups (Poushter et al., 2018). As such, the correlations among social media use, volunteering, and donating cannot be generalized to a larger body of citizens (Hooghe et al., 2010).

The age differences matter, because differences in participation in offline activities are well recognized, but we do not know if similar differences exist in the online context. If similar age differences are evident in the online version of activities, then we might conclude the digitalization of these activities has reproduced old inequalities in participation (Ackermann & Manatschal, 2018). Ackermann and Manatschal (2018) examine online and offline volunteering and find age is positively related to offline volunteering but negatively related to online volunteering. More generally, they find different variables predict the different modes of these activities, challenging the resource models of participation that dominate the literature on offline civic and political engagement.

I do not presuppose that social media are more effective with respect to donating as opposed to volunteering. However, in considering whether the effects may differ, I point out distinct features of these two activities. The differences between donating and volunteering may impact how social media are used to connect with donors versus volunteers. While donating involves a large number of people contributing small amounts of money or goods (Borst et al., 2017), volunteering tends to involve a small number of people contributing large amounts of time (Reed & Selbee, 2001). As such, social media may be used differently. Social media may be important for mobilizing and sustaining the involvement of these volunteers. In contrast, social media use may be important for expanding reach to thousands with hopes of gathering small contributions from many people. Nonetheless, little research has been done on how social media use is connected to donating and volunteering activities.

### Donating

Donating is any contribution to a charitable group or organization. The contribution could be monetary or an in-kind contribution, such as food or clothing. Monetary donations are particularly interesting, given they could be facilitated through social media, a website, or even through mobile messaging (Martin, 2015). Crowd-sourcing initiatives have transformed online donations. Crowdfunding platforms, such as GoFundMe, allow users to create their own pages and raise money for personal initiatives. Social media can enhance the effectiveness of these crowdsourcing campaigns (see literature review in Borst et al., 2017).

Stoycheff et al. (2017) reviewed a decade’s worth of research on social media in six key academic journals (663 articles) and coded the content of these studies. They find less than 0.6% of scholarship examines social media use for crowdsourcing and fundraising. One of these studies (Saxton & Wang, 2014) focuses on Facebook Causes to examine charitable donations as per Internal Revenue Source 990 forms; however, this feature is no longer offered on Facebook as this platform is continually changing.

Through social media, people can initiate pleas for donations to charities but can also re-circulate solicitations from civic organizations (Saxton & Wang, 2014). Social media can establish a social norm of giving to charity (Einolf, 2011). Sharing an announcement of one’s donation can lead to additional donations from one’s peer network (Waddingham, 2013). Indeed, social media can help exert peer pressure to donate (Borst et al., 2017). For example, Boulianne et al. (2018) examine tweets in relation to a community disaster and find widespread calls to donate to support displaced community members. These tweets were widely “liked.” Survey data support the correlation between following news about this disaster on social media and subsequent helping (Boulianne et al., 2018). The findings support an online network effect related to caring and helping.

On the other hand, there is a fear that a call to donate or volunteer circulated on social media can produce a bystander effect where no one acts because they assume others will (Borst et al., 2017). Examining 10 case studies of crowdsourcing, Borst et al. (2017) ends with a caution that requests for financial assistance should be clearly stated and limited in number to be effective. My first set of research questions (RQ1) is:

**Research Question 1 (RQ1):** How does following nonprofit groups on social media relate to donating to nonprofit organizations and groups?

**Research Question 1a (RQ1a):** Does this relationship differ by social media platform (Facebook, Instagram, Twitter)?

**Research Question 1b (RQ1b):** Does this relationship differ for online donating compared to offline donating?

### Volunteering

Volunteering is defined as unpaid work for not-for-profit groups oriented toward solving a community problem or helping people in need (Musick & Wilson, 2008). The Internet could be a valuable resource for recruiting new volunteers (Emrich & Pierdzioch, 2016) but could also have negative impacts on volunteering (Filsinger & Freitag, 2019). Using time use measures as part of a survey of more than 7000 Swiss people, Filsinger & Freitag, 2019 find a negative impact on volunteering. Because volunteering involves time and energy, hours spent online may detrimentally impact volunteering (Filsinger & Freitag, 2019). Vilhelmson et al. (2017) examine time use data from 3000 Swedes. They find time spent online takes time away from a variety of activities, such as one’s job, taking care of one’s kids, eating, traveling, and talking to friends, but does not affect volunteering hours. These studies do not consider the possibility that time spent online might be for volunteer work. Online volunteering could involve activities such as building websites, responding to distress messages, translating documents, and helping with social media campaigns (see examples in Ackermann & Manatschal, 2018).

Social media could be used to broadcast volunteer positions to the community. I believe that ‘followers’ of community organizations’ social media profiles are prime targets for recruitment given their pre-existing interest in the organization, i.e., standby citizens (Amnå & Ekman, 2014). Further, they may share these posts with their own network, expanding the organizations’ reach for recruitment, as observed in relation to donating. Again, this sharing of calls to action could create a social norm of volunteering. Social media may be particularly effective with respect to volunteering because donating is an individualistic activity, whereas volunteering can involve working as a group. In the context of offline volunteering, this group work could involve work on a fundraising event, such as a fun run; in the context of online volunteering, this group work might involve compiling content for a newsletter. Social media may be particularly effective for group-based civic activities, compared to more individualistic activities, because social media support social ties and interaction.

Other studies argue that the impacts of digital media on volunteering depend on the type of use. Leisure uses of the Internet had minimal impact on donating and volunteering for the German Red Cross, but using the German Red Cross website had positive and significant effects (Emrich & Pierdzioch, 2016). Erhardt & Freitag, 2021 have longitudinal data about digital media use and participation in civic organizations. They find negative effects when considering online games and civic membership, but positive effects when considering email and participation in civic organizations. Piatak and Mikkelsen (2021) consider social media use and volunteering for nonprofit organizations; they find a significant positive effect. Indeed, of their 23 predictors of formal volunteering, social media use, education, ideology, and religiosity are the only significant predictors. As such, the degree to which digital media, or specific social media platforms, are used for social activities or recreation would have differing impacts on volunteering. My second set of research questions is:

**Research Question 2 (RQ2):** How does following nonprofit groups on social media relate to volunteering for nonprofit organizations and groups?

**Research Question 2a (RQ2a):** Does this relationship differ by social media platform (Facebook, Instagram, Twitter)?

**Research Question 2b (RQ2b):** Does this relationship differ for online volunteering compared to offline volunteering?

### Data

The survey data were gathered online from September to November 2019, after receiving ethics approval from MacEwan University in June 2019 (File No. 101,662). In total, almost 6300 people responded to the survey: 1700 from the United States, 1542 from the United Kingdom, 1510 from France, and 1539 from Canada. The survey questions were administered by Kantar to an online panel. Quotas were used to ensure the sample characteristics matched the census profile (age, gender) for each of the four countries. Table 1 outlines these descriptive statistics for the pooled sample. Within each country, the age, education, and gender distribution are similar to the official statistics, indicating representative survey data (see Supplementary Material for a comparison of official statistics and sample characteristics). The data and replication files are available at https://doi.org/10.6084/m9.figshare.17019623.v1

**Table 1.** Descriptive Statistics for Variables Used in the Analysis.

Variable	Percentage	Valid responses
Dependent variables		
Offline donating	42.8	6286
Online donating	28.1	6284
Offline volunteering	28.5	6290
Online volunteering	20.0	6285
Independent variables		
Follows nonprofits on Facebook	14.1	6291
Follows nonprofits on Twitter	6.1	6291
Follows nonprofits on Instagram	5.6	6291
Control variables		
Age 18–24	10.4	6291
Age 25–34	16.8	6291
Age 35–44	16.2	6291
Age 45–54	17.2	6291
Age 55 and up	39.3	6291
Female = 1	51.2	6263
High school or less	48.5	6291
Lower college	18.4	6291
Bachelor’s degree	23.1	6291
More than a bachelor’s degree	10.0	6291
Income, quintile 1	18.9	5871
Income, quintile 2	19.5	5871
Income, quintile 3	24.1	5871
Income, quintile 4	20.1	5871
Income, quintile 5	17.4	5871

### Dependent Variables

For donating, respondents were first prompted: “For the following questions, we want to know what you do offline only. Please exclude online activities, we will ask about those activities next. In the past 12 months, have you donated money to a nonprofit or charity organization (like an environmental organization or Red Cross)?”. Then, they were asked about donating “money *online* to a nonprofit or charity organization (like an environmental organization or Red Cross)” in the past 12 months. Approximately 42.8% of respondents (*n* = 6286) had donated offline and 28.1% had donated online in the past year.

For volunteering, respondents were asked, “In the past 12 months, have you volunteered offline for a nonprofit organization or charity (like an environmental organization or Red Cross)?”. Respondents were offered the response options: never, rarely, time to time, and often. Given the infrequency of this activity and to enable a comparison to donating (which is dichotomous), I dichotomized this variable. In the Supplementary Material, I offer a robustness test of the original variable, using multinomial logistic regression. The key distinction is between those who never volunteer and those who volunteer, justifying a focus on binary logistic regression. The responses were subsequently recoded into never (0) versus all other responses (1). Approximately 28.5% of respondents (*n* = 6290) had volunteered offline at least once in the past year. The survey then asked, “in the past 12 months, have you volunteered online for a nonprofit Forganization or charity (like an environmental organization or Red Cross)?”. Approximately 20.0% of respondents (*n* = 6285) had volunteered online. Based on these results, offline forms of volunteering are more popular than online forms. As mentioned, little research has been conducted on online forms of volunteering for nonprofit organizations.

### Independent Variables

Participants were asked how often they used specific social media platforms. While eight platforms were listed, I offered an exhaustive set of follow-up questions to people who self-identified as users of Facebook (74.3%), Instagram (39.8%), or Twitter (38.6%). These platforms are among the most popular sites among the profile-based social networking sites (Perrin & Andersen, 2019). These users were asked the following: “During the last 12 months, have you followed a nonprofit organization or charity (like an environmental organization or Red Cross)?”. If they were non-users of the platform, they were coded as zero. As such, for the whole sample, I found that 14.1% of the sample followed a nonprofit on Facebook, 6.1% on Twitter, and 5.6% on Instagram. Respondents were more likely to establish a tie to a civic group on Facebook compared to other platforms.

### Control Variables

We controlled for gender, age, education, and household income, as these variables affect volunteering and donating (Bekkers & Wiepking, 2011; Bourassa & Stang, 2016; Reed & Selbee, 2001). Gender was measured using male (0) and female (1). Age was reported in years, but converted into a series of dummy (age group) variables for analysis to examine the possible non-linear effects of age on volunteering and donating. Education was measured as a series of dummy variables: high school or less, some college, bachelor’s degree, and more than a bachelor’s degree.

The final variable used in this study is household income. Income was originally coded in intervals and in the relevant currency for the respective country. These categories were adjusted to create quintiles placing approximately 20% of the sample in each category. These categories were converted into a series of dummy variables for analysis. Because all independent and dependent variables are dichotomous, I used logistic regression for analysis.

## Results

Before outlining the findings for the research questions, I highlight results for the key variable—following nonprofits on social media platforms (Table 2), as little research is available on this topic. The findings illustrate the problem with youth-focused samples and offer important context for subsequent analysis, when I use “following nonprofits” as a predictor. Compared to other age groups, young people (18–24 years) are more likely to follow nonprofits on all social media platforms. The specific pattern of age effects depends on the platform, but one result is consistent: compared to 18–24 year olds, other age groups are less likely to follow nonprofits on social media platforms. Females are more likely than males to follow nonprofits on Facebook and Instagram, but no gender differences are apparent on Twitter. Education is a strong and positive predictor of following nonprofits on all social media platforms; income is not a significant predictor. The models perform best in explaining civic uses of Instagram compared to the other two platforms (see Nagelkerke R-square = .111).

**Table 2.** Logistic Regression Analysis of Following Nonprofits on Social Media Platforms.

	Model 1 follows nonprofits on Facebook		Model 2 follows nonprofits on Twitter		Model 3 follows nonprofits on Instagram	
	Exp (B)	*p*-value	Exp (B)	*p*-value	Exp (B)	*p*-value
Age 25–34	0.882	.359	0.577	.002	0.656	.011
Age 35–44	0.794	.103	0.613	.006	0.507	<.001
Age 45–54	0.689	.010	0.471	<.001	0.280	<.001
Age 55 and up	0.580	<.001	0.219	<.001	0.108	<.001
Female = 1	1.451	<.001	0.839	.118	1.378	.008
Some college	1.505	<.001	1.616	.003	1.443	.025
Bachelor’s degree	1.646	<.001	2.211	<.001	1.442	.017
More than a bachelor’s degree	1.918	<.001	2.795	<.001	2.582	<.001
Income, quintile 2	1.171	.202	1.278	.164	0.750	.118
Income, quintile 3	1.027	.825	0.815	.261	0.557	.002
Income, quintile 4	1.083	.527	0.915	.632	0.862	.409
Income, quintile 5	1.093	.501	1.001	.996	0.896	.561
Nagelkerke R-square	.034		.065		0.111	
Valid n	5844		5844		5844	

Reference group for age is 18–24 years, for gender is male, for education is high school or less, and for income is the lowest quintile.

For the first research question, I examine how following nonprofit groups on social media impact donating to nonprofit organizations and groups. Across all platforms, I find strong positive relationships between following nonprofits and online and offline donating (Table 3). The odds ratios (Exp (B)) range from 1.454 to 3.432. The strength of the relationship depends on the platform (RQ1a) and for online versus offline forms of donating (RQ1b).

**Table 3.** Logistic Models of Donating.

	Model 1 OFFLINE Exp (B)	*p*-value	Model 2 ONLINE Exp (B)	*p*-value
Follows nonprofits on Facebook	3.432	<.001	2.792	<.001
Follows nonprofits on Twitter	1.454	.007	2.042	<.001
Follows nonprofits on Instagram	1.708	<.001	1.657	<.001
Age 25–34	1.011	.928	0.756	.021
Age 35–44	1.104	.407	0.707	.005
Age 45–54	1.302	.025	0.644	<.001
Age 55 and up	2.680	<.001	0.874	.214
Female = 1	0.909	.097	0.880	.042
Some college	1.197	.020	1.176	.060
Bachelor’s degree	1.509	<.001	1.614	<.001
More than a bachelor’s degree	1.751	<.001	2.179	<.001
Income, quintile 2	1.234	.025	1.192	.097
Income, quintile 3	1.409	<.001	1.269	.018
Income, quintile 4	1.774	<.001	1.472	<.001
Income, quintile 5	2.206	<.001	2.094	<.001
Nagelkerke R-square	0.158		0.136	
Valid *n*	5840		5838	

Reference group for age is 18–24 years, for gender is male, for education is high school or less, and for income is the lowest quintile.

In relation to platform-specific effects, I find that following a nonprofit on Facebook has a much stronger relationship with online and offline donating compared to following a nonprofit on Twitter or Instagram (Table 3). Following a nonprofit on Facebook more than triples the odds of donating offline to a nonprofit organization (Exp (B) = 3.432). In terms of online, there is a slightly smaller, but still very strong relationship. Following a nonprofit on Facebook almost triples the odds of donating online to a nonprofit organization (Exp (B) = 2.792). Following a nonprofit on Instagram has a consistent effect on offline donating and online donating (Exp (B) = 1.708, 1.657). Finally, I find the odds of donating online double if one follows a nonprofit on Twitter. Unlike Facebook, Twitter’s effects are larger for online donating compared to offline donating (Exp (B) = 2.042 vs. 1.454). In sum, for Research Question 1a, I find Facebook stands out as a platform having the strongest relationship with donating. For Research Question 1b, Facebook has a larger impact on offline donating, whereas Twitter has a larger impact on online donating. Finally, Instagram has a consistent impact on both modes of donating.

Education and income positively impact both online and offline donating. Females are slightly less likely to donate online, but no significant gender differences are apparent for offline donating. Finally, age impacts donating online versus offline; older people are more likely to donate offline, whereas younger people are more likely to donate online. The models perform equally well in predicting online and offline forms of donating (see Nagelkerke R-square: .136 and .158, respectively).

For the second research question, I examine how following nonprofit groups on social media relates to volunteering for nonprofit organizations and groups (Table 4). I find that following nonprofits on social media has a strong, positive relationship with volunteering. The odds ratios range from 1.590 to 2.896. However, I see differences by platform (RQ2a) and by mode (online vs. offline) (RQ2b).

**Table 4.** Logistic Models of Volunteering.

	Model 1 OFFLINE Exp (B)	*p*-value	Model 2 ONLINE Exp (B)	*p*-value
Follows nonprofits on Facebook	2.896	<.001	2.316	<.001
Follows nonprofits on Twitter	1.590	<.001	1.877	<.001
Follows nonprofits on Instagram	2.110	<.001	2.381	<.001
Age 25–34	0.630	<.001	0.627	<.001
Age 35–44	0.543	<.001	0.530	<.001
Age 45–54	0.354	<.001	0.277	<.001
Age 55 and up	0.349	<.001	0.126	<.001
Female = 1	0.771	<.001	0.599	<.001
Some college	1.197	.035	0.984	.876
Bachelor’s degree	1.494	<.001	1.416	<.001
More than a bachelor’s degree	1.957	<.001	1.727	<.001
Income, quintile 2	1.103	.337	1.048	.686
Income, quintile 3	1.026	.796	0.893	.310
Income, quintile 4	1.155	.157	0.889	.316
Income, quintile 5	1.289	.018	1.056	.654
Nagelkerke R-square	0.150		0.222	
Valid *n*	5844		5839	

^a^Reference group for age is 18–24 years, for gender is male, for education is high school or less, and for income is the lowest quintile.

As observed with donating, Facebook has the strongest impact on offline volunteering compared to both other platforms. Following a nonprofit on Facebook triples the odds of offline volunteering (Exp (B) = 2.896). All of the other platform coefficients are quite consistent in their impact on online and offline volunteering. These coefficients suggest following a nonprofit on social media doubles the odds of both offline and online volunteering. Following a nonprofit on Facebook doubles the odds of online volunteering (Exp (B) = 2.316), and we see similar effect sizes for Instagram (Exp (B) = 2.381) and Twitter (Exp (B) = 1.877). The smallest effect size is for following a nonprofit on Twitter on offline volunteering (Exp (B) = 1.590). Following a nonprofit on Instagram doubles the odds of offline volunteering (Exp (B) = 2.110).

In sum, for Research Question 2a, I find Facebook stands out as a distinctive platform, specifically for offline volunteering but not so for online volunteering (RQ2b). For online volunteering, the platforms have rather consistent effect sizes that suggest following a nonprofit on social media doubles the odds of volunteering. While distinct platform effects on donating online and offline are evident, these platform specificities are less pronounced in relation to volunteering.

Education positively impacts both online and offline volunteering. Income has little impact on online and offline volunteering. Females are less likely to volunteer online and offline. Finally, age has a strong impact on volunteering. Compared to young people, older people are less likely to volunteer online or offline. The models perform better at explaining online volunteering compared to offline volunteering (see Nagelkerke R-square: .222 vs. .150, respectively).

**Figure 1.**
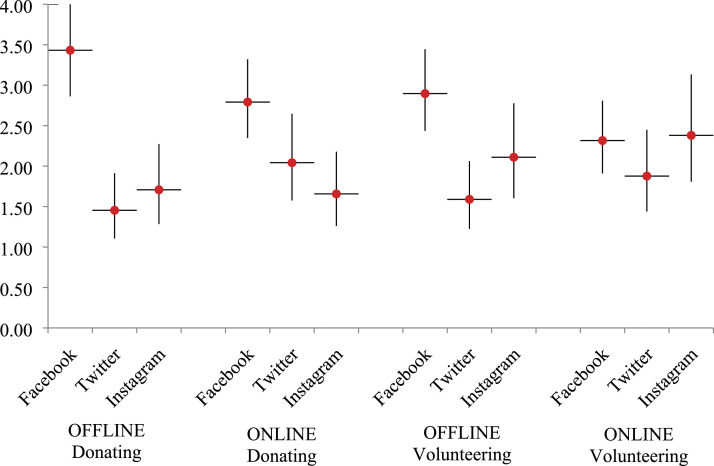
Marginal effects of social media platforms on donating and volunteering.

To summarize the findings about the three key independent variables and the four dependent variables, Figure 1 offers a visual display of the coefficients with 95% confidence intervals. Following nonprofit groups on any social media positively relates to donating (RQ1) and volunteering (RQ2) for nonprofit organizations and groups. Compared to Instagram and Twitter, Facebook is distinctive in relation to offline donating (RQ1a). Facebook has a larger role than Instagram in online donating (RQ1a). Facebook has a larger role than Twitter in offline volunteering (RQ2a). For online volunteering, the platforms have similar roles (RQ2a). As for comparisons between online and offline forms of activities, Instagram’s role in offline donating/volunteering is similar to its role in online donating/volunteering (RQ1b/RQ2b).

## Discussion

Citizens follow nonprofit groups as part of curating their social media feeds (Wells & Thorson, 2017). They are strategically using social media to invite more content from these organizations. Citizens can be viewed as “standby citizens,” who are using social media to stay informed and alert about the organizations that interest them, ready to take action if necessary (Amnå & Ekman, 2014). Nonprofit organizations can post calls to donate or volunteer on their social media profiles. These calls could be circulated through social networks, establishing social norms of giving and volunteering (Borst et al., 2017; Boulianne et al., 2018; Einolf, 2011; Saxton & Wang, 2014; Waddingham, 2013).

The survey data reveal that following nonprofit organizations on social media is positively related to civic engagement. Existing research aggregates volunteering and donating together (or with other forms of civic/political engagement), making it difficult to detect nuances in these forms of civic engagement. In addition, combining these activities would run against scholarship on civic and political participation, which clearly establishes that these activities are distinct from each other and from other forms of participation (Theocharis et al., 2021). Donating is characterized as a low-effort activity that depends on many contributors, while volunteering is a high-effort activity that depends on a handful of dedicated people. In this context, we may expect differences in the role of social media as well as differences in who participates in these activities. Little research has separated the online and offline modes of these activities and my analysis suggests social media platforms differ in their relationships with both forms.

Distinguishing online and offline forms of activities is important, because the potential to mobilize and who engages both differ. For example, I found older people are more likely to donate offline but young people are more likely to engage online. Combining these two modes would blur these important distinctions. Ackermann and Manatschal (2018) explain how these differences in predictors provide evidence to address mobilization versus reinforcement theories of digitalization. If online modes of activities are performed by the same people as offline modes, then that would support the reinforcement theory. Reinforcement theories will lead to larger participation gaps and exacerbate social and political inequality (Oser & Boulianne, 2020). Instead, online activities could be engaging people who are not engaged offline, offering support for mobilization. I find larger differences in online modes than offline modes. For gender, no differences were evident in offline donating, but men were more likely to donate online compared to women. The role of household income does not differ by mode. Most importantly, age impacts patterns of online and offline participation. Age has a larger impact on online participation, favoring youth engagement and a mobilization process. For offline donating, the age differences favor older people. And, as expected from prior research, youth are more likely to volunteer regardless of the mode (Turcotte, 2015).

My research suggests that current approaches to using Facebook pages are especially effective in mobilizing followers to donate; indeed, following a nonprofit on Facebook triples the odds of donating online and offline. Further research might compare the topic of posts across various platforms to see if the platform differences relate to content. For example, do nonprofits send out calls for donation more often on Facebook than other platforms? Could the content rather than the affordances or size of the user group explain the stronger role of Facebook? As mentioned, Borst et al. (2017) summarize 10 case studies of crowdsourcing and caution about the use of social media for requests for financial assistance. This premise deserves further attention with A/B testing to determine if requests for donations are more or less effective on different platforms.

Finally, this study was not able to establish causal direction. It might not be that following nonprofit organizations on social media causes donating and volunteering, but rather a reverse effect. Erhardt & Freitag, 2021 address causal flow with their longitudinal design and conclude it depends on the type of digital media use, but they do not address platform-specific effects and also consider membership rather than volunteering and donating. Perhaps social media are used exclusively by existing supporters: donating or volunteering leads to following these organizations on social media.

The causal direction is important because the implications are very different. For example, if following civic groups is exclusive to supporters, then the objective may be oriented towards transparency. In other words, donors may follow the organization to determine how their funds are being used. Emrich and Pierdzioch (2016) refer to this activity as a transparency function. Alternatively, for volunteers, following these organizations on social media may have a different purpose. They may follow the organization as a way to illustrate their support, learn about new events or training opportunities, or build their identities as conscientious citizens (Emrich & Pierdzioch, 2016). A longitudinal analysis could demonstrate whether following the social media pages of nonprofit organizations leads to civic engagement or if individuals who are civically engaged are more likely to follow such pages. If the former is true, community organizations hoping to encourage civic engagement could expand their recruitment efforts online. If the latter is true, community organizations could intensify their communication with current ‘followers’ as a means of increasing those followers’ level of civic engagement with the organization. Either of these potential findings would be a substantial addition to the literature on this subject.

This paper makes three distinct contributions to the existing literature about social media use and civic engagement. First, I examine platform-specific effects. Facebook is used more so than other platforms to cultivate ties to civic groups. While following nonprofits on any platform positively correlates with online and offline volunteering, Facebook is distinctive in the magnitude of the relationship. The digital architecture enables civic groups to offer longer posts, promote content, target users, and offer a variety of advertising formats (Bossetta, 2018). However, Facebook shares these features with Instagram, but the two platforms do not have identical relationships with civic engagement. As such, the digital architecture alone cannot explain these outcomes. While Instagram and Facebook offer the same features, the differential roles could be related to how civic organizations use or do not use these platforms. Perhaps the stronger correlations reflect that civic organizations are using the platform affordances of Facebook to offer sophisticated targeting and advertising formats. While these affordances are available on Instagram, civic organizations may not be using these features.

I argue that the size of the Facebook audience is what is important. Facebook is the most popular of the three platforms, which creates an incentive for civic groups to use the platform to find volunteers and donors. Facebook is used more so than other platforms to cultivate ties to civic groups and that Facebook connections have stronger correlations with offline as opposed to online forms of participation. Facebook’s popularity with citizens creates an incentive for civic groups to use this platform to create and maintain their presence on this platform; my research suggests this investment has payoffs in terms of volunteering and donating.

Second, I examine how these connections impact the likelihood of volunteering and donating as separate and distinct activities and their online and offline manifestations, addressing a clear gap related to online participation. I find strong age differences, which suggest caution in extrapolating findings from student and youth samples to a larger population. I continue a line of research by Ackermann and Manatschal (2018), but use a cross-national sample to examine differences in predictors for online and offline participation. I expand on this research by considering the role of income and social media. Income is important for understanding resource differences in patterns of online and offline participation; social media platforms are important for understanding the mechanism of mobilization. I find that income matters for donating, but not volunteering; mode of participation does not influence the role of income.

Third, the survey data are from a large, representative sample from four countries. While cross-national differences are not the focal point (cf. Boulianne, 2019; Boulianne et al., 2019), this cross-national sample offers tests of the relationships between social media use and civic engagement with a diverse sample, again addressing the limits of existing scholarship that tends to be US-focused (Boulianne, 2019; Stoycheff et al., 2017). Across all four countries, following nonprofit organizations on social media is highly correlated with volunteering and donating. I offer a distinct and robust theory of social media and civic engagement to examine engagement across a diverse sample. Social media can be used to cultivate flows (Wells & Thorson, 2017), creating connections between organizations and sympathetic citizens who are on standby (Amnå & Ekman, 2014) and ready to mobilize when called upon. These potential mobilization effects seem most effective in relation to nonprofits’ Facebook pages and for young people.

## Supplemental Material

sj-pdf-1-ssc-10.1177_08944393211067687 – Supplemental Material for Standby Ties that Mobilize: Social Media Platforms and Civic Engagement Click here for additional data file. Supplemental Material, sj-pdf-1-ssc-10.1177_08944393211067687 for Standby Ties that Mobilize: Social Media Platforms and Civic Engagement by Shelley Boulianne in Social Science Computer Review

## References

[bibr1-08944393211067687] Ackermann K. Manatschal A. (2018). Online volunteering as a means to overcome unequal participation? The profiles of online and offline volunteers compared. New Media & Society, 20(12), 4453–4472. 10.1177/1461444818775698

[bibr2-08944393211067687] Amnå E. Ekman J. (2014). Standby citizens: Diverse faces of political passivity. European Political Science Review, 6(2), 261–281.

[bibr3-08944393211067687] Atkinson L. (2015). Buying in or tuning out: The role of consumption in politically active young adults. In Gil de Zuniga H. (Ed.), New technologies and civic engagement: New agendas in communication (pp. 23–45). Routledge.

[bibr4-08944393211067687] Bekkers R. Wiepking P. (2011). A literature review of empirical studies of philanthropy: Eight mechanisms that drive charitable giving. Nonprofit and Voluntary Sector Quarterly, 40(5), 924-973. 10.1177/0899764010380927.

[bibr5-08944393211067687] Blank G. Lutz C. (2017). Representativeness of social media in Great Britain: Investigating Facebook, LinkedIn, Twitter, Pinterest, Google plus, and Instagram. American Behavioral Scientist, 61(7), 741–756. 10.1177/0002764217717559

[bibr6-08944393211067687] Borst I. Moser C. Ferguson J. (2017). From friendfunding to crowdfunding: Relevance of relationships, social media, and platform activities to crowdfunding performance. New Media & Society, 20(4), 1–19. 10.1177/1461444817694599 PMC6256715 30581357

[bibr7-08944393211067687] Bossetta M. (2018). The digital architectures of social media: Comparing political campaigning on Facebook, Twitter, Instagram, and Snapchat in the 2016 U.S. election. Journalism & Mass Communication Quarterly, 95(2), 471–496. 10.1177/1077699018763307

[bibr8-08944393211067687] Boulianne S. (2015). Social media use and participation: A meta-analysis of current research. Information, Communication & Society, 18(5), 524-538. 10.1080/1369118x.2015.1008542.

[bibr9-08944393211067687] Boulianne S. (2019). Revolution in the making? Social media effects across the globe. Information, Communication & Society, 22(1), 39–54. 10.1080/1369118X.2017.1353641

[bibr10-08944393211067687] Boulianne S. (2020). Twenty years of digital media effects on civic and political participation. Communication Research, 47(7), 947–966. 10.1177/0093650218808186

[bibr11-08944393211067687] Boulianne S. Minaker J. Haney T. J. (2018). Does compassion go viral? Social media, caring, and the Fort McMurray wildfire. Information Communication & Society, 21(5), 697-711. 10.1080/1369118x.2018.1428651.

[bibr12-08944393211067687] Boulianne S. Steen-Johnsen K. Koc-Michalska K. Bimber B. (2019, May). Online media, networks, and offline volunteering: A longitudinal and comparative stud*y**.* [Paper presented at the international communication association annual meeting].

[bibr13-08944393211067687] Bourassa M. Stang A. (2016). Knowledge is power: Why public knowledge matters to charities. International Journal of Nonprofit and Voluntary Sector Marketing, 21, 13–30. 10.1002/nvsm.1537

[bibr14-08944393211067687] Chan M. Chen H. Lee F. L. F. (2016). Examining the roles of mobile and social media in political participation: A cross-national analysis of three Asian societies using a communication mediation approach. New Media & Society, 19(4), 2003-2021. 10.1177/1461444816653190.

[bibr15-08944393211067687] Clement J. (2019). Number of monthly active Instagram users, 2013–2018. Statista. https://www.statista.com/statistics/253577/number-of-monthly-active-instagram-users/

[bibr16-08944393211067687] Einolf C. J. (2011). Gender differences in the correlates of volunteering and charitable giving. Nonprofit and Voluntary Sector Quarterly, 40(6), 1092–1112. 10.1177/0899764010385949

[bibr17-08944393211067687] Emrich E. Pierdzioch C. (2016). The internet and the commitment of volunteers: Empirical evidence for the Red Cross. Nonprofit and Voluntary Sector Quarterly, 45(5), 1-18. 10.1177/0899764015624980.

[bibr18-08944393211067687] Erhardt J. Freitag M. (2021). The Janus-Face of digitalization: The relation between internet use and civic engagement reconsidered. Social Science Computer Review, 39(3), 315-334. 10.1177/0894439319861966.

[bibr19-08944393211067687] Filsinger M. Freitag M. (2019). Internet use and volunteering: Relationships and differences across age and applications. Voluntas: International Journal of Voluntary and Nonprofit Organizations, 30, 87-97. 10.1007/s11266-018-0045-4.

[bibr20-08944393211067687] Hooghe M. Stolle D. Maheo V. Vissers S. (2010). Why can’t a student be more like an average person? Sampling and attrition effects in social science field and laboratory experiments. The Annals of the American Academy of Political and Social Science, 628(1), 85-96. 10.1177/0002716209351516.

[bibr21-08944393211067687] Kahne J. Lee N. Feezell J. T. (2013). The civic and political significance of online participatory cultures among youth transitioning to adulthood. Journal of Information Technology & Politics, 10(1), 1-20. 10.1080/19331681.2012.701109.

[bibr22-08944393211067687] Kim Y. Khang H. (2014). Revisiting civic voluntarism predictors of college student’s political participation in the context of social media. Computers in Human Behavior, 36(July), 114-121. 10.1016/j.chb.2014.03.044.

[bibr23-08944393211067687] Martin J. A. (2015). Mobile news use and participation in elections: A bridge for the democratic divide? Mobile Media & Communication, 3(2), 230-249. 10.1177/2050157914550664.

[bibr24-08944393211067687] Musick M. Wilson J. (2008). Volunteers: A social profile. Indiana University Press.

[bibr25-08944393211067687] Newman N. Fletcher R. Schulz A. Andi S. Nielson R. K. 2020. Digital news report 2020. Reuters Institute, University of Oxford. http://www.digitalnewsreport.org

[bibr26-08944393211067687] Oser J. Boulianne S. (2020). Reinforcement effects between digital media use and political participation: A meta-analysis of repeated-wave panel data. Public Opinion Quarterly, 84(S1), 355–365. 10.1093/poq/nfaa017

[bibr27-08944393211067687] Pasek J. More E. Romer D. (2009). Realizing the social internet? Online social networking meets offline civic engagement. Journal of Information Technology & Politics, 6(3-4), 197-215. 10.1080/19331680902996403.

[bibr28-08944393211067687] Perrin A. Andersen M. (2019). Share of U.S. adults using social media, including Facebook, is mostly unchanged since 2018. Pew Research Center. https://www.pewresearch.org/fact-tank/2019/04/10/share-of-u-s-adults-using-social-media-including-facebook-is-mostly-unchanged-since-2018/

[bibr29-08944393211067687] Piatak J. Mikkelsen I. (2021). Does social media engagement translate to civic engagement offline? Nonprofit and Voluntary Sector Quarterly, 1-23. 10.1177/0899764021999444.

[bibr30-08944393211067687] Poushter J. Bishop C. Chwe H. (2018). Social network adoption varies widely by country. Pew Research Center. https://www.pewresearch.org/global/2018/06/19/3-social-network-adoption-varies-widely-by-country/

[bibr31-08944393211067687] Reed P. B. Selbee L. K. (2001). The civic core in Canada: Disproportionality in charitable giving, volunteering, and civic participation. Nonprofit and Voluntary Sector Quarterly, 30(4), 761-780. 10.1177/0899764001304008.

[bibr32-08944393211067687] Russmann U. Svensson J. (2016). Studying organizations on Instagram. Information, 7(4), 1–12. 10.3390/info7040058

[bibr33-08944393211067687] Saxton G. D. Wang L. (2014). The social network effect: The determinants of giving through social media. Nonprofit and Voluntary Sector Quarterly, 43(5), 850-868. 10.1177/0899764013485159.

[bibr34-08944393211067687] Stoycheff E. Liu J. Wibowo K. A. Nanni D. P. (2017). What have we learned about social media by studying Facebook? A decade in review. New Media & Society, 19(6), 1-13. 10.1177/1461444817695745.

[bibr35-08944393211067687] Theocharis Y. de Moor J. van Deth J. W. (2021). Digitally networked participation and lifestyle politics as new modes of political participation. Policy and Internet, 13(1), 30-53. 10.1002/poi3.231.

[bibr36-08944393211067687] Turcotte M. (2015). Volunteering and charitable giving in Canada (Catalogue no. 89-652-X2015001). Statistics Canada. http://www.statcan.gc.ca/pub/89-652-x/89-652-x2015001-eng.pdf

[bibr37-08944393211067687] Valenzuela S. Park N. Kee K. F. (2009). Is there social capital in a social network site? Facebook use and college students’ life satisfaction, trust, and participation. Journal of Computer-Mediated Communication, 14(4), 875–901. 10.1111/j.1083-6101.2009.01474.x

[bibr38-08944393211067687] Vilhelmson B. Thulin E. Ellder E. (2017). Where does time spent on the Internet come from? Tracing the influence of information and communications technology use on daily activities. Information, Communication & Society, 20(2), 250-263. 10.1080/1369118x.2016.1164741.

[bibr39-08944393211067687] Waddingham J. (2013). The future of Facebook fundraising. International Journal of Nonprofit and Voluntary Sector Marketing, 18, 187-191. 10.1002/nvsm.1460.

[bibr40-08944393211067687] Warren R. Wicks R. H. (2011). Political socialization: Modeling teen political and civic engagement. Journalism & Mass Communication Quarterly, 88(1), 156-175. 10.1177/107769901108800109.

[bibr41-08944393211067687] Wells C. Thorson K. (2017). Combining big data and survey techniques to model effects of political content flows in Facebook. Social Science Computer Review, 35(1), 33-52. 10.1177/0894439315609528.

[bibr42-08944393211067687] Wicks R. H. LeBlanc Wicks J. Morimoto S. A. Maxwell A. Schulte S. R. (2013). Correlates of political and civic engagement among youth during the 2012 presidential campaign. American Behavioural Scientist, 58(5), 622-644. 10.1177/0002764213515226.

[bibr43-08944393211067687] Xenos M. Vromen A. Loader B. D. (2014). The great equalizer? Patterns of social media use and youth political engagement in three advanced democracies. Information, Communication & Society, 17(2), 151-167. 10.1080/1369118X.2013.871318.

[bibr44-08944393211067687] Xie W. (2014). Social network site use, mobile personal talk and social capital among teenagers. Computers in Human Behavior, 41(December), 228-235. 10.1016/j.chb.2014.09.042.

