# Supplementary material for: Standby Ties that Mobilize: Social Media Platforms and Civic Engagement
Source: Soc Sci Comput Rev. 2022 Feb 21;41(3):1001–16. doi: 10.1177/08944393211067687 (PMC10240620; doi:10.1177/08944393211067687)
Supplement: sj-pdf-1-ssc-10.1177_08944393211067687 – Supplemental Material for Standby Ties that Mobilize: Social Media Platforms and Civic Engagement [file sj-pdf-1-ssc-10.1177_08944393211067687.pdf]

APPENDIX 1 COMPARISON OF SURVEY AND OFFICIAL STATISTICS

		USA	UK	France	Canada
1 18-24	Official	12%	11%	10%	11%
	Survey	11%	11%	10%	9%
2 25-34	Official	18%	17%	15%	16%
	Survey	18%	17%	15%	17%
3 35-44	Official	16%	16%	16%	16%
	Survey	16%	16%	16%	17%
4 45-54	Official	17%	18%	17%	18%
	Survey	17%	18%	17%	17%
5 55+	Official	37%	37%	42%	39%
	Survey	38%	37%	42%	40%
0 Male	Official	49%	51%	49%	49%
	Survey	48%	51%	49%	46%
1 Female	Official	51%	49%	51%	51%
	Survey	52%	49%	51%	53%

USA

Age and sex (2017): Age in Entire U.S. for 2017 American Community Survey

UK

Sex and Age (2016):

https://www.ons.gov.uk/peoplepopulationandcommunity/populationandmigration/populationestimates/bulletins/annualmidyearpopulationestimates/mid2016#main-points

France

Age and sex (2018):

https://www.insee.fr/en/statistiques/2382609?sommaire=2382613

Canada

Age groups and gender (2016):

https://www12.statcan.gc.ca/datasets/Index-eng.cfm?Temporal=2016&Theme=115&VNAMEE=&GA=-1&S=0

		US	UK	France	Canada
1 BASIC STUDIES	Official	42%	56%	56%	45%
	Survey	40%	56%	57%	42%
2 ADVANCED STUDIES	Official	58%	44%	44%	55%
	Survey	60%	44%	43%	58%

US

Education (2015):

https://www.census.gov/data/tables/2015/demo/education-attainment/p20-578.html

UK

Education (2011):

https://www.ons.gov.uk/employmentandlabourmarket/peopleinwork/employmentandemployeetypes/bulletins/keystatisticsandquickstatisticsforlocalauthoritiesintheunitedkingdom/2013-12-04

France

Education (June 2017): https://publication.enseignementsup-recherche.gouv.fr/eesr/10EN/EESR10EN_ES_20-level_of_education_among_the_general_population_and_among_young_people.php

Canada

Education (2017):

https://www12.statcan.gc.ca/datasets/Index-eng.cfm?Temporal=2017&Theme=123&VNAMEE=&GA=-1&S=0

APPENDIX 2 MULTINOMIAL LOGISTIC REGRESSION OF OFFLINE VOLUNTEERING

	Never n=4132		Rarely n=664		Sometimes n=649	
	Exp(B)	p-value	Exp(B)	p-value	Exp(B)	p-value
Follows nonprofits on Facebook	0.228	<.001	0.513	<.001	0.625	.003
Follows nonprofits on Twitter	0.648	.026	1.015	.947	1.061	.782
Follow nonprofits on Instagram	0.344	<.001	0.625	.033	0.699	.096
Age 25 to 34	1.417	.127	0.789	.333	1.004	.988
Age 35 to 44	1.127	.589	0.521	.007	0.604	.040
Age 45 to 54	1.855	.008	0.612	.055	0.600	.054
Age 55 and up	1.057	.782	0.212	<.001	0.373	<.001
Female=1	1.154	.202	0.865	.275	0.859	.255
Lower college	1.080	.639	1.367	.099	1.399	.081
Bachelor’s degree	0.645	.002	0.845	.312	1.077	.655
More than a bachelor’s degree	0.519	<.001	0.823	.362	1.258	.271
Income, Quintile 2	0.883	.507	0.981	.930	0.953	.828
Income, Quintile 3	0.956	.803	0.942	.783	1.011	.960
Income, Quintile 4	0.898	.573	1.135	.568	0.968	.883

Income, Quintile 5	0.600	.007	0.773	.248	0.652	.057
Nagelkerke R-square	.148					
Valid n	5844					

Reference group for age is 18 to 24 years, for gender is male, for education is high school or less, for income is the lowest quintile, and for offline volunteering is “often” (n=399).

APPENDIX 3 MULTINOMIAL LOGISTIC REGRESSION OF ONLINE VOLUNTEERING

	Never n=4631		Rarely n=546		Sometimes n=469	
	Exp(B)	p-value	Exp(B)	p-value	Exp(B)	p-value
Follows nonprofits on Facebook	0.457	<.001	1.012	.957	1.149	.532
Follows nonprofits on Twitter	0.365	<.001	0.593	.037	0.669	.109
Follow nonprofits on Instagram	0.260	<.001	0.558	.021	0.549	.018
Age 25 to 34	1.310	.256	0.770	.310	0.823	.460
Age 35 to 44	1.574	.061	0.699	.177	0.939	.812
Age 45 to 54	3.345	<.001	1.012	.967	0.798	.462
Age 55 and up	8.516	<.001	1.172	.606	0.981	.951
Female=1	1.940	<.001	1.260	.192	1.112	.555
Lower college	1.591	.073	1.791	.036	1.511	.152

Bachelor’s degree	0.690	.056	0.894	.605	1.070	.759
More than a bachelor’s degree	0.548	.014	0.796	.410	1.111	.702
Income, Quintile 2	0.761	.259	0.705	.192	0.854	.570
Income, Quintile 3	1.274	.346	1.065	.822	1.301	.362
Income, Quintile 4	1.444	.179	1.209	.525	1.509	.177
Income, Quintile 5	0.726	.207	0.705	.213	0.767	.359
Nagelkerke R-square	.197					
Valid n	5839					

Reference group for age is 18 to 24 years, for gender is male, for education is high school or less, for income is the lowest quintile , and for online volunteering is “often” (n=193).
